# *DoRWA3* from *Dendrobium officinale* Plays an Essential Role in Acetylation of Polysaccharides

**DOI:** 10.3390/ijms21176250

**Published:** 2020-08-28

**Authors:** Can Si, Jaime A. Teixeira da Silva, Chunmei He, Zhenming Yu, Conghui Zhao, Haobin Wang, Mingze Zhang, Jun Duan

**Affiliations:** 1 Key Laboratory of South China Agricultural Plant Molecular Analysis and Genetic Improvement, South China Botanical Garden, Chinese Academy of Sciences, Guangzhou 510650, China; cans2013@163.com (C.S.); hechunmei26@163.com (C.H.); zhenming311@scbg.ac.cn (Z.Y.); zhaoconghui@scbg.ac.cn (C.Z.); wanghaobin17@scbg.ac.cn (H.W.); zhangmingze@scbg.ac.cn (M.Z.); 2 College of Life Sciences, University of Chinese Academy of Sciences, Beijing 100049, China; 3 Independent researcher, P.O. Box 7, Miki-cho Post Office, Ikenobe 3011-2, Miki-cho, Kita-gun, Kagawa-ken 761-0799, Japan; jaimetex@yahoo.com

**Keywords:** acetyl groups, *Dendrobium officinale*, *REDUCED WALL ACETYLATION*, endoplasmic reticulum

## Abstract

The acetylation or deacetylation of polysaccharides can influence their physical properties and biological activities. One main constituent of the edible medicinal orchid, *Dendrobium officinale*, is water-soluble polysaccharides (WSPs) with substituted *O*-acetyl groups. Both *O*-acetyl groups and WSPs show a similar trend in different organs, but the genes coding for enzymes that transfer acetyl groups to WSPs have not been identified. In this study, we report that REDUCED WALL ACETYLATION (RWA) proteins may act as acetyltransferases. Three *DoRWA* genes were identified, cloned, and sequenced. They were sensitive to abscisic acid (ABA), but there were no differences in germination rate and root length between wild type and *35S::DoRWA3* transgenic lines under ABA stress. Three DoRWA proteins were localized in the endoplasmic reticulum. *DoRWA3* had relatively stronger transcript levels in organs where acetyl groups accumulated than *DoRWA1* and *DoRWA2*, was co-expressed with polysaccharides synthetic genes, so it was considered as a candidate acetyltransferase gene. The level of acetylation of polysaccharides increased significantly in the seeds, leaves and stems of three *35S::DoRWA3* transgenic lines compared to wild type plants. These results indicate that DoRWA3 can transfer acetyl groups to polysaccharides and is a candidate protein to improve the biological activity of other edible and medicinal plants.

## 1. Introduction

Polysaccharides, which are extracted from many edible and medicinal plants, have been widely used in food, cosmetics and pharmaceutical industries due to their therapeutic properties and low toxicity [1,2]. The functional properties of polysaccharides depend on several structural parameters, particularly the composition of monosaccharides, molecular weight and functional groups [3]. Acetyl groups, which are substituted at the backbone or sidechain of polysaccharides, can expose more hydroxyl groups in water, thus influence the solubility, gelation, surface structure and other physical properties of polysaccharides [4,5,6]. Furthermore, in plants, the deacetylation or acetylation of polysaccharides can affect their molecular weight, structure and conformation, and thus influence their biological activity, conferring various activities (antibacterial, antibiofilm, antioxidant, anticoagulant and immunoregulatory) [7,8].

Thus far, three different protein families have been shown to be involved in the *O*-acetylation of polysaccharides, REDUCED WALL ACETYLATION (RWA), ALTERED XYLOGLUCAN9 (AXY9), and TRICHOME BIREFRINGENCE LIKE (TBL), with 4, 1 and 46 members in *Arabidopsis thaliana* [9,10,11,12,13]. The single mutant *rwa2* showed an indistinguishable phenotype and had a 17% lower degree of acetylation (DA) compared with the wild type (WT) [10] while the quadruple mutant *rwa1rwa2rwa3rwa4* displayed a severely dwarfed phenotype and 63% lower DA in rosette leaves [14]. Similarly, *RWA* genes in hybrid aspen downregulated the acetylation of wood, including xylan and xyloglucan, by 15–20%, but this did not affect the height or stem diameter of plants significantly [15]. A single mutant *axy9.2* in *A. thaliana* had smaller leaves and 35% less DA in rosette leaves [11]. The single knockout mutant *tbl27* showed 14% lower DA of xyloglucan, while the double mutant *rwa2tbl27* showed as much as 24% lower DA in rosette leaves [12]. Although the biosynthetic pathway for *O*-acetylation of polysaccharides is fairly clear, very little is known about the mechanism of *O*-acetylation in edible and medicinal plants.

*Dendrobium officinale* Kimura et Migo could be a good model species to address these research challenges. First, as a traditional Chinese medicine (TCM), the in vivo and in vitro biological activities of water-soluble polysaccharides (WSPs), which are the major medicinal ingredients of *D. officinale*, have antioxidant, antitumor, antidiabetic, anti-inflammation, and immunomodulating activities [16]. Secondly, WSPs contain mannose, glucose and acetyl groups substituted at the *O*-2 or *O*-3 site of mannosyl residues [17], and the primary structure, such as mannose, β-(1→4)-Man linkage and acetyl groups, mainly contribute to the bioactivity of WSPs [18]. Finally, the *O*-acetyl content accounts for as much as 2.9% (*w*/*w*, dry weight) of the polysaccharides [19]. Despite this, until now, the mechanism of *O*-acetylation in *D. officinale* had not yet been reported.

This article focuses on *D. officinale RWA* genes. Bioinformatics tools were used to obtain basic information about *DoRWA* genes such as gene structure, *c*is-elements and conserved domains. The expression levels of three *DoRWA* genes in different organs and developmental stages, and in response to abiotic stresses, were also assessed. Three DoRWA proteins were transformed into the protoplasts of *A. thaliana* with a localization marker to assess the localization of these proteins. Most importantly, *35S::DoRWA3* transgenic lines were constructed to verify the biological functions of *DoRWA3*. The exploration of *RWA* genes in this orchid would facilitate the targeting of the genes coding for acetyltransferase.

## 2. Results

### 2.1. Isolation and Sequence Analysis of the DoRWA Genes

Three *DoRWA* genes, named *DoRWA1*, *DoRWA2* and *DoRWA3*, were identified in the *D. officinale* genome [20]. Their open reading frames (ORFs) were 1638, 1638 and 1617 bp long, encoding 545, 545 and 538 aa. The molecular weights (MWs) of the three genes were 63.825, 63.780 and 63.634 Da, and their isoelectric points (pIs) were 8.84, 9.01 and 8.94. The ORF sequences of three genes were submitted to NCBI with the accession numbers MT199223, MT199224 and MT199225. BlastP results revealed that DoRWA1 had 99% similarity with RWA1 of *Dendrobium catenatum* (XP_020674439.1) and 92% similarity with RWA1 of *Phalaenopsis equestris* (XP_020584729.1); DoRWA2 had 99% similarity with RWA1 of *D. catenatum* (XP_028548609.1) and 92% similarity with RWA3L of *P. equestris* (XP_020577546.1); DoRWA3 had 99% similarity with RWA4 of *D. catenatum* (XP_020684246.1) and 84% similarity with RWA4L of *P. equestris* (XP_020573535.1).

### 2.2. Bioinformatics of the DoRWA Genes

**Figure 1:**
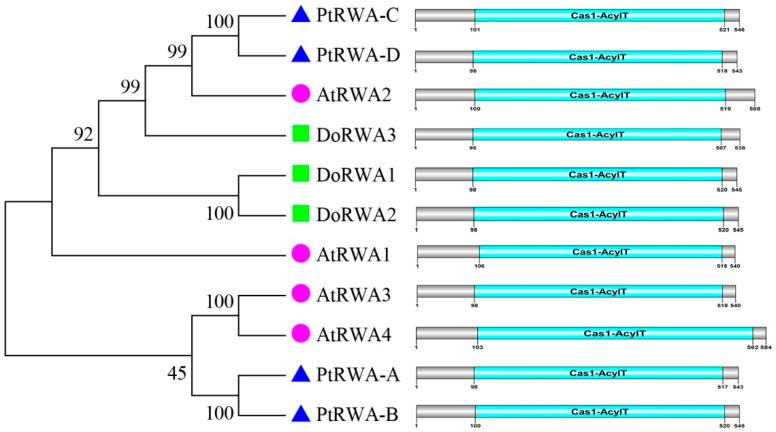
Phylogenetic tree and conserved domain analysis of REDUCED WALL ACETYLATION (RWA) proteins in *Dendrobium officinale*, *Arabidopsis thaliana* and *Populus trichocarpa*. The phylogenetic tree was constructed by the neighbor-joining (N-J) method in MEGA 7.0 software. Domains were drawn by DOG2.0 software. The gene IDs of RWA proteins are: DoRWA1 (MT199223), DoRWA2 (MT199224), DoRWA3 (MT199225), AtRWA1 (At5g46340), AtRWA2 (At3g06550), AtRWA3 (At2g34410), AtRWA4 (At1g29890), PtRWA-A (Potri.001g352300), PtRWA-B (Potri.011g079400), PtRWA-C (Potri.010g148500), and PtRWA-D (Potri.008g102300). The blue area indicates the Cas1-AcylT conserved domain.

**Figure 2:**
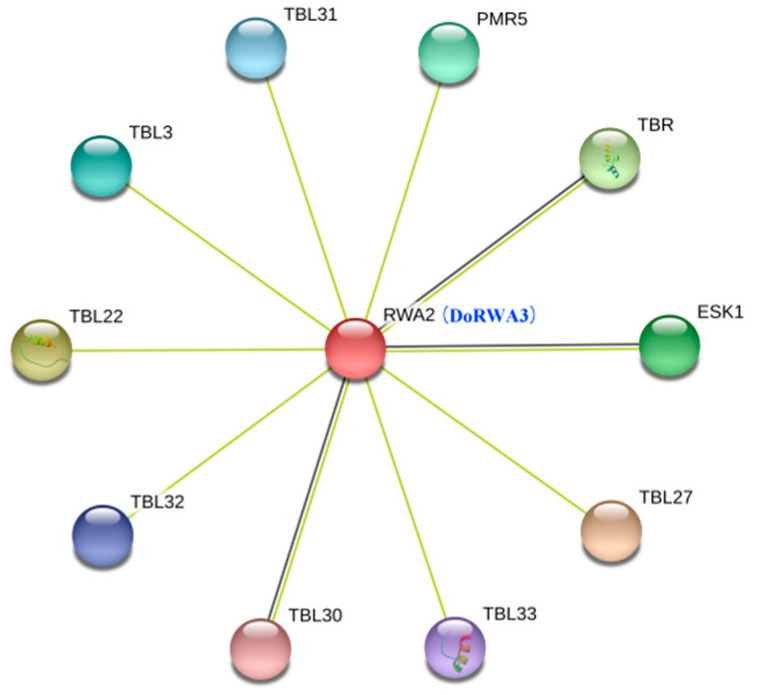
Protein–protein association networks of DoRWA3 using RWA2, a homologous *Arabidopsis thaliana* protein. Yellow lines represent “textmining” and black lines represent “co-expression”.

The RWA protein sequences from three plants were aligned (Figure S1). The highest similarity of these proteins was 71.59%, indicating that RWA was considerably conserved in these plants. These proteins had the same domain Cas1-AcylT (411–459 aa), accounting for 73.77–78.60% of the full length (538–584 aa) (Figure 1). The exon–intron structures and length of the three genes varied. *DoRWA1* (37 kb) and *DoRWA2* (14 kb) had a similar gene structure and contained 16 exons and 15 introns, while *DoRWA3* (6 kb) had 15 exons and 14 introns (Figure S2). The protein sequences of the three plants were also used to construct a phylogenetic tree using the Neighbor-Joining (N-J) method. DoRWA1 and DoRWA2 were clustered as one branch, and DoRWA3 was clustered with AtRWA2, PtRWA-C and PtRWA-D (Figure 1). Using the homologous *A. thaliana* protein, RWA2 was used to conduct a protein–protein interaction network analysis in which RWA2 was correlated with other TBL proteins [12,13] (Figure 2), such as pectin *O*-acetyltransferase TBR [21], xylan *O*-aceryltransferase TBL3 and TBL31 [22], and xyloglucan *O*-acetyltransferase TBL27 [23], indicating that DoRWA2 and DoTBL proteins were active in the same metabolic pathway.

### 2.3. Analysis of cis-Elements, and Expression Patterns of DoRWA Genes under Cold and ABA Treatments

**Figure 3:**
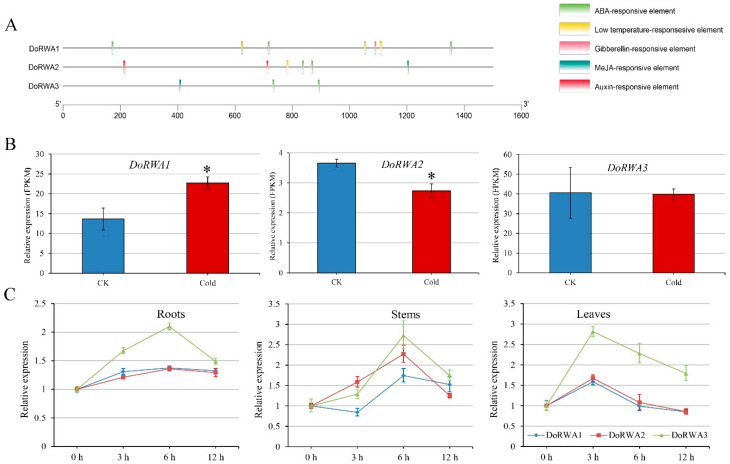
Analysis of *cis*-elements and relative expression levels of three *DoRWA* genes under cold stress (4 °C) and abscisic acid (ABA), (100 μM) treatment. (**A**) Analysis of *cis*-elements in the promoter region of three *DoRWA* genes. Different colors represent different *cis*-elements. (**B**) Relative expression level of three *DoRWA* genes under CK (20 °C) and cold stress (4 °C) using the FPKM value. (**C**) Relative expression level of three *DoRWA* genes in the ABA treatment. The transcript level of *DoRWA* genes at 0 h was set as 1. Each data bar represents mean ± SD (*n* = 3). * indicates *p* < 0.05 between the expression level of three *DoRW*A genes in CK and cold treatment according to Duncan’s multiple range test (DMRT).

The *cis*-elements of the three genes contained hormone-responsive elements (methyl jasmonate, abscisic acid (ABA), auxin, gibberellin) and abiotic stress-responsive elements (low temperature) (Figure 3A). All three *DoRWA* genes had the ABA-responsive element while *DoRWA1* and *DoRWA2* (but not *DoRWA3*) had a low temperature-responsive element. To verify these predictions, the expression patterns of the three *DoRWA* genes in response to cold and ABA were assessed. In the cold treatment, the relative expression level (fragments per kilobase per million, FPKM) of *DoRWA1* was upregulated, *DoRWA2* was downregulated, but *DoRWA3* showed no difference (Figure 3B). In the ABA treatment, the transcript levels of the three genes were upregulated at first, then peaked, but were finally downregulated in three organs (roots, stems, leaves), with peak expression at 6 h in roots and stems, and at 3 h in leaves (Figure 3C).

### 2.4. Cellular Localization of DoRWA Proteins

**Figure 4:**
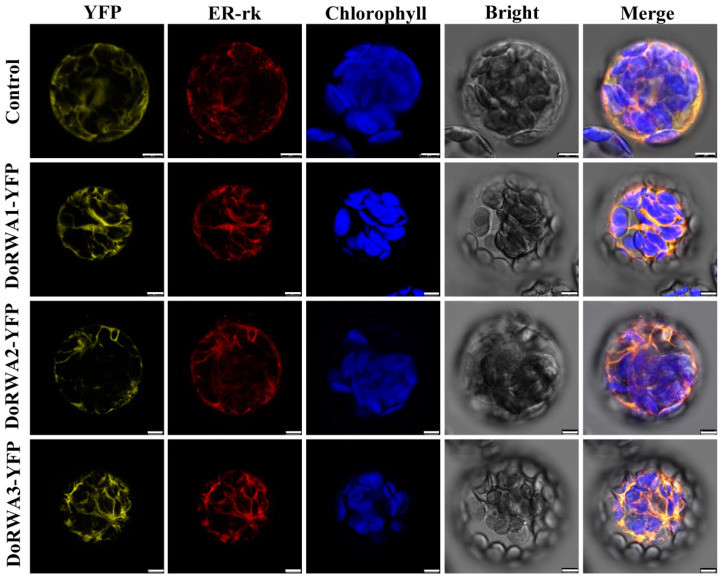
Subcellular localization of YFP, DoRWA1-YFP, DoRWA2-YFP and DoRWA3-YFP with the endoplasmic reticulum localization marker ER-rk in the *Arabidopsis thaliana* mesophyll protoplasts. Bar = 5 μm.

The three DoRWA proteins (DoRWA1, DoRWA2, DoRWA3) had 10, 10 and 11 transmembrane helices, which indicated they may be localized in a membranous organelle (Figure S3A,B,D). The fluorescent signals showed that all three DoRWA proteins were co-localized with the ER-rk (Figure 4), which is also localized in the endoplasmic reticulum (ER) [24]. Furthermore, the YFP fluorescence of DoRWA3 was not localized in the Golgi apparatus (GA) (Figure S3D). These results indicate that the ER plays an important role in the acetylation of polysaccharides.

### 2.5. WSPs and O-Acetyl Groups Mainly Accumulated in the Stems of D. officinale

**Figure 5:**
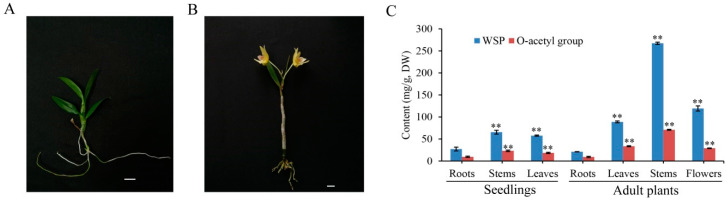
The metabolic accumulation of water-soluble polysaccharides (WSPs) and *O*-acetyl groups in different organs of *Dendrobium officinale*. (**A**) Ten-month-old seedling. Bar = 1 cm. (**B**) Adult plant. Bar = 1 cm. (**C**) The content of WSPs and related *O*-acetyl groups in different organs of seedlings and adult plants. Each data bar represents the mean ± SD (*n* = 3). ** indicates *p* < 0.01 between the content of WSPs and *O*-acetyl groups in the roots of seedlings and in other organs of seedlings and adult plants according to DMRT.

WSPs and *O*-acetyl groups showed a similar trend, accumulating the most in stems, followed by flowers and leaves, and the least in roots of seedlings and adult plants (Figure 5). The content of WSPs in stems was 65.55 mg/g in seedlings and 267.48 mg/g in adult plants. The content of *O*-acetyl groups of WSPs in stems was 23.22 mg/g in seedlings and 70.96 mg/g in adult plants.

### 2.6. Expression Patterns of DoRWA Genes in Different Organs of Seedlings and Adult Plants

**Figure 6:**
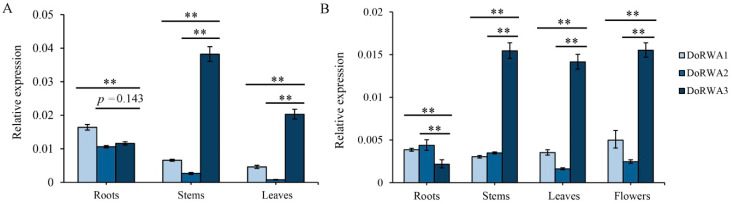
Expression profiles of *DoRWA* genes in different organs. Relative expression levels of three *DoRWA* genes in different organs of (**A**) seedlings and (**B**) adult plants. In A and B, *EF-1α* served as the control. Each data bar represents the mean ± SD (*n* = 3). ** indicates *p* < 0.01 between the expression level of *DoRWA3* and *DoRWA1* (*DoRWA2*) according to DMRT.

In *D. officinale* seedlings, the expression profiles of *DoRWA1* and *DoRWA2* were similar, with higher transcript levels in roots than in stems and leaves, while the relative expression of *DoRWA3* was higher in stems than in roots and leaves (Figure 6A). In adult plants, the expression levels of *DoRWA1* and *DoRWA2* showed fewer differences in several organs, but the expression of *DoRWA3* was relatively higher in stems, leaves and flowers where acetyl groups accumulated (Figure 6B). The mRNA ratio of *DoRWA3* was 5.80- and 14.41-fold higher than *DoRWA1* and *DoRWA2* in stems, and 4.42- and 25.94-fold higher than in leaves of seedlings (Figure 6A). Similar results were also found in adult plants (Figure 6B).

### 2.7. Co-Expression of DoRWA3 with Synthetic Genes of Polysaccharides

**Figure 7:**
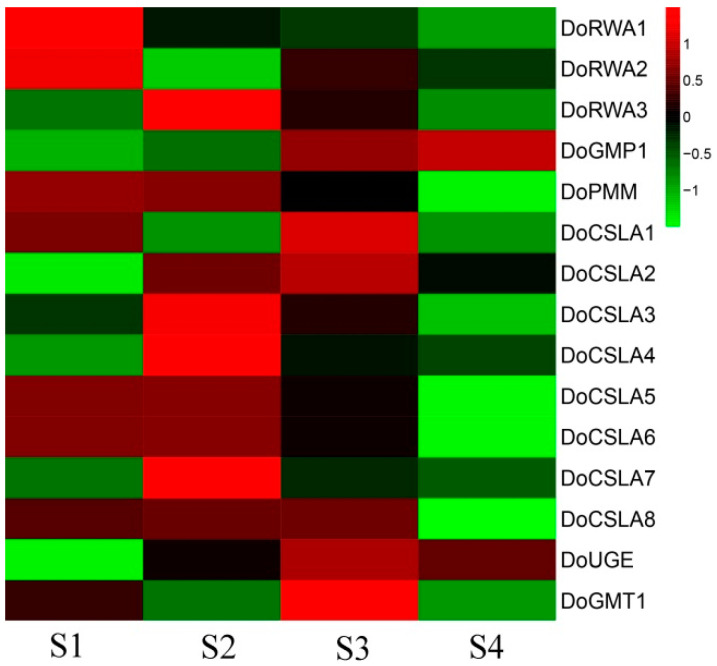
Expression profiles of three *DoRWA* genes and other polysaccharide-related genes in four developmental stages. RPKM values of different genes were log-transformed (log of mean RPKM).

In the four developmental stages, the content of WSPs increased from S1 to S3, decreased from S3 to S4, and peaked at S3 [25]. The transcript levels of key genes related to polysaccharides in *D. officinale*, such as *cellulose synthesis-like* (*CSL*) [25], *GDP-mannose pyrophosphorylation* (*GMP*) [26], *UDP glucose 4-epimerase* (*UGE*) [27] and *GDP-mannose transporter* (*GMT*) [28] peaked at S2 or S3, corresponding to trends in the content of WSPs (Figure 5C). The expression profile of *DoRWA3* peaked at S2, and showed a close association with the key genes described above (Figure 7), This indicates that *DoRWA3* may be a candidate gene responsible for coding the enzyme that transfers acetyl groups to WSPs.

### 2.8. DoRWA3 Overexpression Increased the Acetylation Level of Polysaccharides in A. thaliana

**Figure 8:**
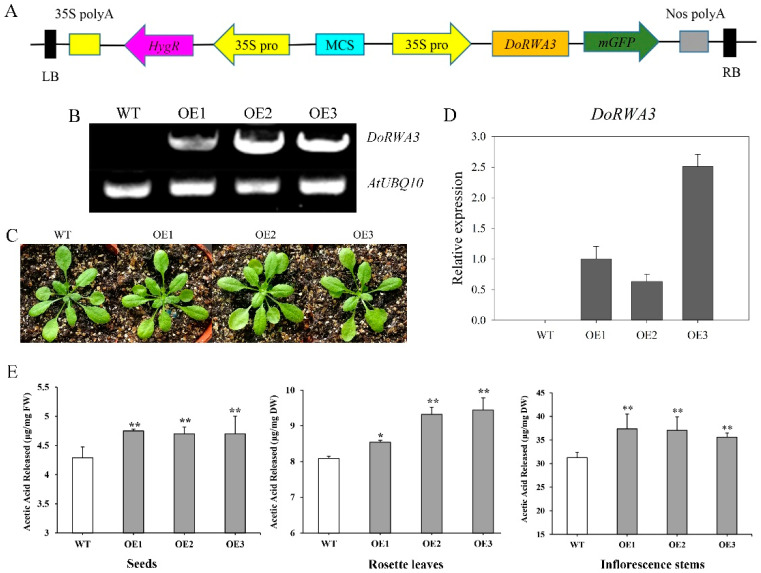
Overexpression of *DoRWA3* increased the acetylation level of polysaccharides in *A. thaliana*. (**A**) The pCAMBIA1302 vector used for *A. thaliana* transformation. (**B)** Semi–quantitative PCR of the *DoRWA3* gene in wild type (WT) and three overexpression (OE) transgenic (*35S::DoRWA3*) lines: OE1, OE2 and OE3. (**C**) Phenotype of the WT and three OE transgenic lines. (**D**) qRT-PCR analysis of *DoRWA3* in the WT and three OE transgenic lines. (**E**) The content of released acetic acid in different organs of the WT and three OE transgenic lines. The transcript level of *DoRWA3* in OE1 was set as 1. FW, fresh weight; DW, dry weight. Data bars represent the mean ± SD (*n* = 3). * and ** indicate *p* < 0.05 and *p* < 0.01 between the WT and OE transgenic lines according to DMRT.

**Figure 9:**
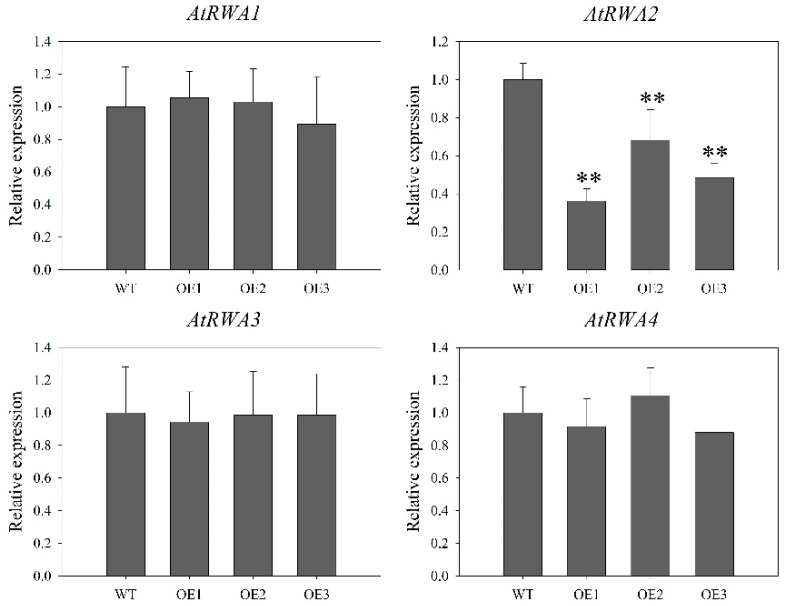
Relative expression levels of four *AtRWA* genes in the wild type (WT) and three overexpression (OE) *35S::DoRWA3* transgenic lines (OE1, OE2, OE3). The transcript level of *AtRWA* genes in the WT was set as 1. Each data bar represents mean ± SD (*n* = 3). ** indicate *p* < 0.01 between the transcript levels of three *DoRWA* genes WT and OE transgenic lines according to DMRT.

The semi-quantitative PCR and qRT-PCR results (Figure 8B,D) indicated that *DoRWA3* was successfully inserted into the *A. thaliana* genome and could be transcribed normally. There were no differences in the phenotype (color, size, flowering time, etc.) between WT and the three overexpression (OE) transgenic lines (Figure 8C). Simultaneously, the transcript level of four *AtRWA* genes in WT and three OE transgenic lines were also tested. There were no differences in the relative expression levels of *AtRWA1*, *AtRWA3*, and *AtRWA4* between WT and transgenic lines, but the expression level of *AtRWA2* was lower in the three transgenic lines compared with WT (Figure 9).

In seeds, the content of released acetic acid was about 1.11-, 1.10- and 1.10-fold higher in the three transgenic OE lines than in WT (Figure 8E). The corresponding values were 1.06-, 1.15- and 1.17-fold higher in rosette leaves (Figure 8E) and 1.20-, 1.19- and 1.14-fold higher in inflorescence stems (Figure 8E). The exogenously inserted *DoRWA3* gene increased the level of acetylation of polysaccharides in seeds, leaves and stems of transgenic *A. thaliana* by 10–11%, 6–17%, and 14–20%, respectively. The released acetic acid of stems (31–38 mg/g) was higher than leaves (8–9.5 mg/g) and seeds (4–5 mg/g), indicating that stems accumulated more *O*-acetyl groups in polysaccharides of *A. thaliana*, relative to *D. officinale*.

### 2.9. ABA Sensitivity Was Not Affected by Constitutive Expression of DoRWA3

The germination rate of WT and transgenic lines was almost 100% (Figure S4A,C). ABA treatment reduced root length of all plants: in WT, root length decreased from 5.95 cm (control) to 5.11 cm (2 μM ABA), but there were no significant (*p* > 0.05) differences between the WT and transgenic plants (Figure S4D). These findings indicate that ABA sensitivity may not be affected by the exogenous *DoRWA3*.

## 3. Discussion

Acetyl groups affect the biological activities of polysaccharides in many edible and medicinal plants, such as *Cyclocarya paliurus* [29], *Dendrobium huoshanense* [30], *D. officinale* [31] and *Plantago asiatica* [32], *Amorphophallus konjac* [33] and *Aloe vera* [34]. Konjac glucomannan (KGM), which is extracted from the corm of *A. konjac* [35], consisting of β-1,4-linked mannose and glucose residues (molar ratio: 1.6:1) with substituted acetyl groups. Deacetylated KGM has less health benefits than KGM [36]. Acemannan extracted from *A. vera* leaves contains the β-1,4-linked mannose and glucose (molar ratio: 3:1) with substituted acetyl groups [37]. After treating with alkaline to remove acetyl groups, the solubility, hydrophilicity and bioactivities of acemannan are reduced [5].

Cell wall acetylation has been shown to play broad roles in plant abiotic and biotic responses. *rwa2-1* and *rwa2-3* mutants showed enhanced resistance to the fungus *Botrytis cinerea* [10]. Some genes related to hormones and oxidative stress, such as auxin, ABA, jasmonic acid (JA), cytokinin (CK), light, cold and drought are down or upregulated in the transcriptome of mutant *rwa2-3* compared with the WT, but no difference in the phenotype (growth and root length) was observed when the WT and *rwa2-3* were treated with hormones (auxin, ABA, JA and CK) [38]. In this study, many *cis*-elements in the promoter region of *DoRWA* genes were associated with hormones and abiotic stress (Figure 3A). qRT-PCR proved that *DoRWA* genes were responsive to ABA (Figure 3C), but there were no differences in germination rate and root length between *A. thaliana* WT and OE transgenic lines when treated with ABA (Figure S4), indicating that *DoRWA3* might not participate in the ABA-dependent signaling pathway.

The expression levels of four *RWA* genes in *A. thaliana* varied in different organs, but they all had relatively higher expression levels in inflorescence stems than in leaves and flowers, while *AtRWA2* expression was also relatively higher in leaves than in flowers [10]. The expression levels of four *RWA* genes in *Populus tremula* varied in different organs (seeds, roots, leaves, buds and flowers) and treatment (drought): *PtRWA-A* was always expressed more than *PtRWA-B*, and *PtRWA-C* more than *PtRWA-D* [15]. In different organs, *DoRWA1* and *DoRWA2* showed the highest mRNA ratio in roots where the content of acetyl groups was lowest compared with other organs (Figure 6A), most likely because they had similar gene structures and conserved domains (Figure 1 and Figure S2); *DoRWA3* had relatively higher expression levels in organs where acetyl groups accumulated, namely stems, leaves and flowers (Figure 6). The mRNA ratio of *DoRWA3* showed significantly higher expression levels than *DoRWA1* and *DoRWA2* in stems, leaves and flowers (Figure 6). In four developmental stages, *DoRWA3*, but not *DoRWA1* and *DoRWA2*, was co-expressed with the synthetic genes of WSPs (Figure 7). Thus, we hypothesize that *DoRWA3* is a key gene coding for an acetyltransferase, while *DoRWA1* and *DoRWA2* may be redundant.

The AtRWA2 protein was localized in the ER or GA in *Nicotiana benthamiana* leaves or carrot protoplasts [9,10], showing species specificity. Since polysaccharides are synthesized in the GA and the related GDP-mannose transporter protein is also localized in the GA [28], it was expected that the DoRWA proteins would also be localized in the GA. However, they were localized in the ER (Figure 4), indicating that ER may be responsible for the upstream acetylation of polysaccharides.

The phenotype (growth and morphology) of a single mutant was not different from the WT [10], while triple and quadruple *rwa* mutants showed a severely dwarfed phenotype [14]. Similarly, the three *35S::DoRWA3* transgenic lines were indistinguishable from the WT (Figure 8C). This suggests that a single *rwa* mutant or *DoRWA3* OE transgenic lines have no effect on the growth of *A. thaliana*.

A minor reduction in the acetylation level was detected in the inflorescence stems of single mutants *rwa1*, *rwa2*, *rwa3* and *rwa4* [9], indicating that the four AtRWA genes may influence the level of cell wall acetylation in *A. thaliana*. To verify if these four *AtRWA* genes participate in increasing the acetylation level in cell wall polymers, their transcript levels were detected in WT and three OE transgenic lines. The expression patterns of *AtRWA1*, *AtRWA3* and *AtRWA4* were similar, while the expression profile of *AtRWA2* was lower in the three transgenic lines compared with the WT (Figure 9), indicating that the four *AtRWA* genes did not play a vital role in increasing the acetylation level of polysaccharides in OE transgenic lines.

The level of acetylation of cell wall residues from leaves was significantly reduced in a single mutant *rwa2-1* compared with WT, while only a small difference was found in the stems [10]. The quadruple mutant *rwa1rwa2rwa3rwa4* showed the largest reduction of acetylation level, about a 63% decrease [14]. In our study, the content of acetic acid increased significantly in the three transgenic lines compared with the WT in three organs: 6–17% in leaves, 10–11% in seeds and 14–20% in stems (Figure 8E). These findings indicate that exogenous *DoRWA3* could increase the acetylation level of polysaccharides in *A. thaliana*. *DoRWA3* could be considered as a candidate gene to improve the biological activities of polysaccharides in other edible and medicinal plants.

## 4. Materials and Methods 

### 4.1. Plant Materials and Hormone Treatment

The young seedlings of *D. officinale* were cultured on half-strength (macro- and micronutrients) Murashige and Skoog (MS) medium [39], containing 0.5% activated carbon, 2% sucrose, and 0.5% agar (pH 5.7). Adult *D. officinale* plants were planted in a ground bark substrate in a greenhouse of South China Botanical Garden (Guangzhou, Guangdong, China) under ambient conditions. The seeds of *A. thaliana* Columbia (Col-0) were placed at 4 °C and continual darkness for 3 d, then transferred to a substrate containing nutritive soil and vermiculite (*v*/*v*, 2:1) under a controlled environment (80% humidity, 22 °C, 16-h photoperiod). For the ABA treatment, 10-month-old plantlets of *D. officinale* were treated with 100 μM ABA for 0, 3, 6 and 12 h, 0 h was regarded as the control group. The roots, stems, leaves, and flowers of young seedlings and adult plants were collected, frozen in nitrogen liquid, and stored at −80 °C.

### 4.2. RNA Extraction, cDNA Synthesis and qRT-PCR Analysis

Total RNA was extracted by an sodium dodecyl sulfate (SDS) method [40]. Briefly, 0.2 g of fresh sample was ground into a powder with liquid nitrogen. Extraction buffer (100 mM Tris-HCl, pH = 8.0; 50 mM EDTA, pH = 8.0; 500 mM NaCl; 1% SDS; 4% β-mercaptoethanol) was added and vortexed. After centrifuging at 12,000 rpm for 5 min, 1/3 (*v*/*v*) KAC (pH 4.8, 5 mol/L) was added to the solution to remove polysaccharides. After centrifuging at 12,000 rpm for 10 min, the supernatant was washed with chloroform:isoamyl alcohol (*v*/*v*, 24:1) and precipitated by 100% isopropanol. The precipitate was washed twice with 75% ethanol, dissolved in RNAase-free water, and stored at −80 °C. Any contaminating DNA was removed by Recombinant DNAase I (TaKaRa Bio Inc., Dalian, China) following the manufacturer’s protocol.

Purified RNA was reverse transcribed to cDNA by the GoScript^TM^ Reverse Transcription System Protocol (Promega, Madison, WI, USA) according to the manufacturer’s instructions. cDNA was further used for gene cloning and qRT-PCR analysis. For qRT-PCR analysis, the iTaq^TM^ Univeral SYBR^®^ Green Supermix (Bio-Rad Laboratories Co. Ltd., Hercules, CA, USA) was used as the polymerase in the following reaction system: stage 1 (95 °C for 2 min); stage 2 (40 cycles of 95 °C for 15 s, 60 °C for 1 min); stage 3 (95 °C for 15 s, 60 °C for 1 min, 95 °C for 15 s, 60 °C for 15 s). *Actin* (JX294908) and *EF-1α* gene [41] from *D. officinale*, and *Actin2* (At3g18780), *UBC* (At5g25760) and *PP2AA3* (At1g13320) [10] from *A. thaliana* were used as reference genes. The primers designed for qRT-PCR analysis are listed in Table S1. The 2^−ΔΔ*C*T^ method [42] was used to calculate the relative expression levels of different genes. All treatments were sampled as three biological and technical replicates.

### 4.3. Identification and Cloning of DoRWA Genes

Four *A. thaliana* RWA proteins were downloaded from TAIR (https://www.arabidopsis.org/): AtRWA1 (At5g46340), AtRWA2 (At3g06550), AtRWA3 (At2g34410) and AtRWA4 (At1g29890) [10]. They were used as queries to search for homologous proteins in the *D. officinale* protein database [19] using Bioedit software [43]. All putative *D. officinale* RWA proteins were further identified by BlastP in NCBI (https://blast.ncbi.nlm.nih.gov/Blast.cgi) to discard any repeated proteins or proteins without the conserved domains of the RWA family. The identified *RWA* genes were used to design the specific primers for gene cloning.

*RWA* genes were cloned from cDNA using KOD FX polymerase (Toyobo Co. Ltd., Osaka, Japan) with the following protocol: stage 1 (94 °C for 3 min); stage 2 (40 cycles of 98 °C for 10 s, 55 °C for 30 s, 72 °C for 2 min); stage 3 (72 °C for 10 min). PCR products were separated on a 1% agarose gel, purified by a DNA Gel Extraction Kit (Dongsheng Co. Ltd., Guangzhou, China), linked to the PMD-18T vector (TaKaRa Bio Inc.), sequenced by Beijing Genome Institute (Shenzhen, Guangdong, China), then submitted to NCBI. The protein sequences of the three genes were submitted to ExPASy (https://web.expasy.org/protparam/) to calculate MWs and theoretical pIs. The primers designed for cloning the three *DoRWA* genes are listed in Table S1.

### 4.4. Bioinformatics Analysis

The RWA protein sequences from *D. officinale*, *A. thaliana* and *Populus trichocarpa* were initially aligned with DNAMAN 7.0 software (Lynnon Biosoft Crop., San Ramon, CA., USA). To complete a phylogenetic analysis, all RWA proteins from these three plants were further aligned by MUSCLE (https://www.ebi.ac.uk/Tools/msa/muscle/), then were used to construct a Neighbor-Joining (N-J) tree built in MEGA 7.0 software [44] with the following parameters: 1000 bootstrap replications; pair deletion. Protein domain structures were drawn in DOG software [45]. Gene structure, including exons, introns, 5′-UTRs and 3′-UTRs of *DoRWA* genes, were obtained from the *D. officinale* gff database [20], then submitted to GSDS version 2.0 [46] to draw the exon-intron structure. The promoter region (from 0 to −1500 bp) of *DoRWA* genes were obtained from the *D. officinale* scaffold database [20], submitted to PlantCare [47] to discover all *cis*-elements, then the type and number of *cis*-elements were assessed and submitted to TBtools software [48]. STRING Version 11.0 was used to analyze the protein–protein association networks [49].

### 4.5. Protoplast Isolation and Subcellular Localization of DoRWA Proteins

Protoplasts were isolated following the protocol described by Schapire et al. [50]. At first, the enzyme solution was made as follows: 1.5% (*w/v*) cellulase R-10 (Yakult Pharmaceutical Industry Co. Ltd., Tokyo, Japan), 0.3% (*w/v*) macerozyme R-10 (Yakult Pharmaceutical Industry), 20 mM KCl (Mackline, Shanghai, China), 20 mM MES (pH 5.7; Sigma-Aldrich, St. Louis, MO, USA), and 0.4 M mannitol (Mackline). The enzyme solution was warmed to 55 °C for 10 min, then 10 mM CaCl_2_ (Sigma-Aldrich) and 0.1% (*w/v*) bovine serum albumin (Sigma-Aldrich) were added after the solution had cooled down. Next, the lower epidermal surface cell layer was peeled off young leaves of 4- to 5-week-old *A. thaliana* plants by autoclave tape, then leaves were treated with the enzyme solution at 25 °C for 2 h (50 rpm). The resulting harvested protoplasts were washed twice by W5 solution containing 154.5 mM NaCl (Mackline), 125 mM CaCl_2_, 5 mM KCl, 2 mM MES (pH 5.7) and 5 mM glucose (Aladdin, Shanghai, China). Finally, protoplasts were gently resuspended in MMG solution that contained 0.4 mM mannitol, 15 mM MgCl_2_ and 4 mM MES (pH 5.7).

To predict transmembrane helices, the sequences of three DoRWA proteins (DoRWA1, DoRWA2, DoRWA3) sequences were submitted to the TMHMM Server v. 2.0 (http://www.cbs.dtu.dk/services/TMHMM/). The full-length coding sequences of the three *DoRWA* genes (stop codon was removed) were inserted into the pSAT6-EYFP-N1 vector [51] at the *Nco*I site using the In-fusion^®^ HD Cloning Kit (TaKaRa Bio Inc.). Since AtRWA2 was localized in the GA [9] or ER [10], to assess in which organelle DoRWA proteins were localized, recombinant protein combined with GA or ER localization marker [24] were transformed into leaf mesophyll protoplasts of 4- to 5-week-old *A. thaliana* plants using PEG-mediated transformation [52]. After maintaining protoplasts at 22 °C for 16 h in the dark, fluorescence signals were visualized with a Leica TCS SP8 STED 3x microscope (Leica Camera AG, Solms, Germany). The primers designed for pSAT6-EYFP-N1-DoRWAs construction are listed in Table S1.

### 4.6. Content of Water-Soluble Polysaccharides and O-Acetyl Groups in Different Organs

The content of WSPs in different organs (roots, stems, leaves) was measured according to He et al. [25]. The *O*-acetyl groups of WSPs were detected by a modified colorimetric method, as described by Gudlavalleti et al. [53]. Briefly, 0.1 g of each organ was weighed accurately, added to 25 mL of purified water, then warmed at 80 °C for 2 h. After centrifuging at 8000 rpm for 10 min, 1 mL of supernatant was added to 2 mL of freshly formulated alkaline hydroxylamine (mixture of 2 mol/L of hydroxylamine hydrochloride and 3.5 mol/L sodium hydroxide (*v*/*v*, 1:1)), then vortexed immediately. After 4 min, 1 mL of 4 mol/L hydrochloric acid and 1 mL of 0.37 mol/L ferrous chloride–hydrochlolic acid were added. Absorbance of the mixture was measured with an ultraviolet spectrophotometer (UV-1800PC; AOE Instruments Co. Ltd., Shanghai, China) at 540 nm. The acetylcholine chloride was used as the standard. In the control, hydrochloric acid was added before the formulated alkaline hydroxylamine.

### 4.7. RNA-Seq Expression Analysis at Four Developmental Stages and under Cold Stress

To develop the expression profiles of *DoRWA* genes and key genes related to the synthesis of WSPs at four developmental stages (S1–S4: WSP content was upregulated from S1–S3, peaked at S3, and downregulated at S4) [25], four raw reads (SRR1917040, SRR1917041, SRR1917042, SRR1917043) [25] corresponding to the S1, S2, S3 and S4 stage, were mapped to the transcriptome sequence database of *D. officinale* [54]. From the mapped database [24], the reads per kilobase per million reads (RPKM) [55] values of three *DoRWA* genes and WSP synthetic genes at S1–S4 stage were first downloaded then were log-transformed to render data suitable for heatmap analysis. To obtain the expression patterns of *DoRWA* genes under cold stress, six raw reads (SRR3210613, SRR3210621, SRR3210626, SRR3210630, SRR3210635, SRR3210636) [56] were downloaded from NCBI, then mapped to the *D. officinale* genome sequence database [20]. The FPKM value was used for gene expression analysis.

### 4.8. Generation of 35S::DoRWA3 Transgenic Lines

The full-length coding sequence of *DoRWA3* (stop codon was removed) was linked with the pCAMBIA1302 vector at the *Nco*I site using the In-fusion^®^ HD Cloning Kit (TaKaRa Bio Inc.). Recombinant plasmid was transformed into *Agrobacterium tumefaciens* EHA105. Inflorescences of 5- to 6-week-old WT plants were transfected by *A. tumefaciens* with the floral dip method [57]. Three homologous OE lines (OE1, OE2, OE3) were screened on half-strength MS (1/2 MS) medium containing 25 μg/mL hygromycin B (Roche Holding AG, Basel, Switzerland). The primers designed for the pCAMBIA1302-DoRWA3 construction are listed in Table S1.

### 4.9. Semi-Quantitative RT-PCR

Total RNAs from the leaves of 1-month-old WT and three OE transgenic lines were extracted and purified as described above. The semi-quantitative PCR reaction was catalyzed by KOD FX polymerase (Toyobo Co. Ltd.) with the following protocol: stage 1 (94 °C for 3 min); stage 2 (40 cycles of 98 °C for 10 s, 56 °C for 30 s, 72 °C for 2 min); stage 3 (72 °C for 10 min). *UBQ10* (At4g05320) from *A. thaliana* was used as the control. PCR products were visualized on a 1% agrose gel under ultraviolet light. The primers designed for semi-quantitative PCR are listed in Table S1.

### 4.10. Cell Wall Preparation and Determination of Acetyl Esters

The rosette leaves and inflorescence stems of 6-week-old plants, and seeds of WT and three *35S::DoRWA3* transgenic lines, were collected then dried at 80 °C for 12 h (seeds were naturally air-dried for 2 weeks). Samples were ground into power for cell wall extraction.

The alcohol-insoluble residues (AIR) of samples were extracted according to Harholt et al. [58]. Briefly, 30 mg of sample was weighed accurately, washed with 1 mL of 96% ethanol, then kept at 70 °C for 30 min to deactivate enzymes. After centrifuging the mixture at 10,000 g for 5 min, the supernatant was removed, 1 mL of 70% ethanol was added, and the mixture was vortexed. The pellet was washed with 70% ethanol, then centrifuged at 10,000 g for 5 min, and this was repeated until the solution became colorless. Finally, the precipitate was washed in 1 mL of 100% acetone, vortexed immediately, placed at room temperature (RT) for 10 min, then centrifuged at 10,000 g for 5 min. The supernatant was discarded and the pellet was oven-dried at 50 °C until constant weight.

AIR (4 mg) of different samples were accurately weighed, saponified by 400 μL of 1 mol/L NaOH, then centrifuged at 150 rpm overnight (at 28 °C). The solution was neutralized with 400 μL of 1 mol/L HCl, then centrifuged at 12,000 rpm for 10 min. The released acetic acid content in the supernatant was determined by using the Acetic Acid Assay Kit (Megazyme, Wicklow, Ireland) as described by Gill et al. [59]. Briefly, 40 μL of supernatant of different samples was added into a UV- capable 96-well plate, then diluted with 64 μL of ddH_2_O. An amount of 42 μL of mixture (Soulution 1 and Solution 2; *v/v*, 2.5:1) was transferred into each sample, mixed and incubated at RT for 3 min. Absorbance of the mixture was read at 340 nm (A_0_). An amount of 12 μL of 10-fold diluted solution 3 was added to the wells, mixed and incubated at RT for 4 min, then read at 340 nm (A_1_). Finally, 12 μL of 10-fold diluted Solution 4 was added to the plate, mixed thoroughly and incubated at RT for 12 min, then read at 340 nm (A_2_). Solution 5 (acetic acid solution) served as the standard. In the control, 40 μL of supernatant was replaced by 40 μL of ddH_2_O. A_0_, A_1_ and A_2_ values were used to calculate ΔA according to the manufacturer’s recommendations.

### 4.11. ABA Treatment and Phenotype Assay

To study the effect of ABA on germination rates, the seeds (48 seeds per sample) of WT and three OE transgenic lines (OE1, OE2 and OE3) were sown on the 1/2 MS medium containing 0 or 1 μM ABA for 4 d. For the root length assay, seedlings growing on 1/2 MS medium for 3 d were transplanted to 1/2 MS medium supplemented with 1 or 2 μM ABA. After vertical culture for 7 d, root length was assessed using ImageJ software (http://rsbweb.nih.gov/ij/).

### 4.12. Statistical Analysis

All data were plotted in Excel 2013 (Microsoft Inc., Redmond, WA, USA) and Sigmaplot 12.0 (Systat Software Inc., San Jose, CA, USA). Data were analyzed by one-way analysis of variance (ANOVA) and means separated by Duncan’s multiple range test (DMRT) (*p* < 0.05, *p* < 0.01) in SPSS version 22.0 software (IBM Corp., Armonk, NY, USA).

## 5. Conclusions

Three *DoRWA* genes, named *DoRWA1*, *DoRWA2* and *DoRWA3*, were cloned from the medicinal orchid, *D. officinale*. Phylogenetic analysis revealed that DoRWA3 was clustered with the identified acetyltransferase genes (i.e., AtRWA2, PtRWA-C, PtRWA-D) into one branch. Interestingly, the *cis*-elements of the three *DoRWA* genes had the ABA-responsive element and their expression patterns were sensitive to ABA treatment. The results of subcellular localization showed that the three DoRWA proteins were localized in the ER, and not in the GA. The *O*-acetyl groups shared a similar trend as WSPs in different organs. qRT-PCR and RNA-seq results showed that *DoRWA3* was mainly expressed in the organs where the *O*-acetyl groups accumulated, displaying significantly higher expression than *DoRWA1* and *DoRWA2* in different organs, except for roots. *DoRWA3* was co-expressed with key genes related to the synthesis of WSPs, so it is regarded as a candidate gene that codes for an acetyltransferase. The acetylation level of polysaccharides in seeds, leaves and stems of the three *A. thaliana* OE transgenic lines was significantly higher than in WT, indicating that *DoRWA3* has a similar function as *AtRWA2*.

## Supplementary Materials

Supplementary materials can be found at https://www.mdpi.com/1422-0067/21/17/6250/s1. Figure S1: Alignment of amino acid sequences of RWA proteins in *Dendrobium officinale*, *Arabidopsis thaliana* and *Populus trichocarpa*; Figure S2: Gene structure of three *DoRWA* genes. Figure S3: Predictions of subcellular localization of DoRWA1 (A), DoRWA2 (B), DoRWA3 (C) and subcellular localization of DoRWA3-YFP with Golgi apparatus localization marker G-rk (D); Figure S4: Comparison of germination rate and root length between wild type (WT) and overexpression (OE) transgenic plants; Table S1: Primers designed for PCR.

## Abbreviations

ABA	Abscisic acid
AIR	Alcohol-insoluble residue
Axy9	Altered xyloglucan9
CSL	Cellulose synthesis-like
CK	Cytokinin
DA	Degree of acetylation
DMRT	Duncan’s multiple range test
ER	Endoplasmic reticulum
FPKM	Fragments per kilobase per million
GMP	GDP-mannose pyrophosphorylation
GMT	GDP-mannose transporter
GA	Golgi apparatus
JA	Jasmonic acid
KGM	Konjac glucomannan
MW	Molecular weight
MS	Murashige and Skoog
N-J	Neighbor-Joining
ORF	Open reading frame
OE	Overexpression
PMM	Phosphomannomutase
pI	Isoelectric Points
RPKM	Reads per kilobase per million
RT	Room temperature
TCM	Traditional Chinese medicine
qRT-PCR	Quantitative real time polymerase chain reaction
RWA	Reduced wall acetylation
TBL	Trichome birefringence-like
UGE	UDP glucose 4-epimerase
WSP	Water-soluble polysaccharide
