# Supplementary material for: DoRWA3 from Dendrobium officinale Plays an Essential Role in Acetylation of Polysaccharides
Source: Int J Mol Sci. 2020 Aug 28;21(17):6250. doi: 10.3390/ijms21176250 (PMC7503274; doi:10.3390/ijms21176250)
Supplement: Supplementary file 1 [file ijms-21-06250-s001.zip › Supplementary table.docx]

**Table S1**. Primers designed for PCR

| Gene | Type | Primer sequences |
| --- | --- | --- |
| Full coding sequence cloning | | |
| *DoRWA1* | F | ATGGTAGAATCGGGTCCCTTAA |
|  | R | TCATGCCGCAGGTATTGGTATC |
| *DoRWA2* | F | ATGGTGGATTCCGGTCCCTTAA |
|  | R | TTATGCCGTGGGTGTAGGTAGG |
| *DoRWA3* | F | ATGGCTACTTCGACAAAAATAA |
|  | R | TCACAGAAGCTTAAGCAACAAC |
| pSAT6-EYFP-N1-DoRWA construction | | |
| *DoRWA1* | F | CGAACGATAGCCATGGAGATGGTAGAATCGGGTCCC |
|  | R | TGAGTCCGGACCATGGTTGCCGCAGGTATTGGTATCT |
| *DoRWA2* | F | CGAACGATAGCCATGGTGATGGTGGATTCCGGTCCC |
|  | R | TGAGTCCGGACCATGGTTGCCGTGGGTGTAGGTAGGA |
| *DoRWA3* | F | CGAACGATAGCCATGGTTATGGCTACTTCGACAAAA |
|  | R | TGAGTCCGGACCATGGTCAGAAGCTTAAGCAACAACA |
| pCAMBIA1302-DoRWA3 construction | | |
| *DoRWA3* | F | GGACTCTTGACCATGGTTATGGCTACTTCGACAAAA |
|  | R | GTCAGATCTACCATGGTCAGAAGCTTAAGCAACAACA |
| semi-quantitative PCR | | |
| *DoRWA3* | F | GGACTCTTGACCATGGTTATGGCTACTTCGACAAAA |
|  | R | GTCAGATCTACCATGGTCAGAAGCTTAAGCAACAACA |
| *AtUBQ10* | F | CGGATCAGCAGAGGCTTATT |
|  | R | GGTGGACTCCTTCTGGATATTG |
| qRT-PCR analysis | | |
| *DoRWA1* | F | CACTTGGTATCGCCAACAAATAC |
|  | R | ACACCAGGTACTTCCCAAATC |
| *DoRWA2* | F | GGGATGGATGCAGGTCTTATT |
|  | R | CGAATCCAGTCATCCAGACATAG |
| *DoRWA3* | F | CCTGTACTTCCTGCTCATCATT |
|  | R | CTCTTCGGTCTGATGCCTATTC |
| *AtRWA1* | F | CGGGATACCCTATGCTCAATTTC |
|  | R | CCGTCTTGAGTGTGTTGGTTAG |
| *AtRWA2* | F | CGGGATCTCTTTCTGTTCCTTTA |
|  | R | TCCTCGGTTTGATGCCTATTC |
| *AtRWA3* | F | GGCGTTTAGCTGCATTATTCTC |
|  | R | GAAGATACCAAGGGCTCCATAC |
| *AtRWA4* | F | GTCTATGGAGCCCTTGGTATTT |
|  | R | CTCCAGGAATCTCCCACATAAG |
| *DoActin* | F | TCCCAAGGCAAACAGAGAAA |
|  | R | GGCCACTAGCATATAGGGAAAG |
| *DoEF-1α* | F | TCAGGCTGACTGTGCTGTCCT |
|  | R | GTGGTGGCGTCCATCTTGTT |
| *Actin2* | F | TCTTCCGCTCTTTCTTTCCAAGC |
|  | R | ACCATTGTCACACACGATTGGTTG |
| *UBC* | F | CTGCGACTCAGGGAATCTTCTAA |
|  | R | TTGTGCCATTGAATTGAACCC |
| *PP2AA3* | F | TAACGTGGCCAAAATGATGC |
|  | R | GTTCTCCACAACCGCTTGGT |
