# Supplementary material for: DoRWA3 from Dendrobium officinale Plays an Essential Role in Acetylation of Polysaccharides
Source: Int J Mol Sci. 2020 Aug 28;21(17):6250. doi: 10.3390/ijms21176250 (PMC7503274; doi:10.3390/ijms21176250)
Supplement: Supplementary file 1 [file ijms-21-06250-s001.zip › Supplementary figures.docx]

**Figure S1.** Alignment of amino acid sequences of RWA proteins in *Dendrobium officinale*, *Arabidopsis thaliana* and *Pterocarpus trichocarpa*. Clustal X2 was used to align the sequences, and the aligned sequences were used to draw the picture by DNAMAN software. The genes IDs are DoRWA1 (MT199223), DoRWA2 (MT199224), DoRWA3 (MT199225), AtRWA1 (At5g46340), AtRWA2 (At3g06550), AtRWA3 (At2g34410), AtRWA4 (At1g29890), PtRWA-A (Potri.001g352300), PtRWA-B (Potri.011g079400), PtRWA-C (Potri.010g148500), and PtRWA-D (Potri.008g102300). The amino acid sequence between two red lines indicates the Cas1-AcylT conserved domain.

**Figure S2**. The gene structure of three *DoRWA* genes. The black square represents an exon, the black line represents an intron, and the blue square represents a UTR.

**Figure S3**. Predictions of subcellular localization of DoRWA1 (A), DoRWA2 (B), DoRWA3 (C) and subcellular localization of DoRWA3-YFP with Golgi apparatus localization marker G-rk (D). Bar = 5 μm.

**Figure S4**. Comparison of germination ratio and root length between wild type (WT) and overexpression (OE) transgenic plants. (A) Phenotype of germination. Seeds were cultured on 1/2 Murashige and Skoog (MS) medium with 0 or 1 μM abscisic acid (ABA) for 4 d. (B) Phenotype of root length. Seedlings were cultured on 1/2 MS medium for 3 d, then transplanted to 1/2 MS medium supplemented with 1 or 2 μM ABA for another 7 d. (C) The germination assay. (D) The root length assay. OE1, OE2, OE3 represent three *35S:DoRWA3* transgenic lines. Each data bar represents mean ± SD (n = 3). Bar = 1.5 cm.


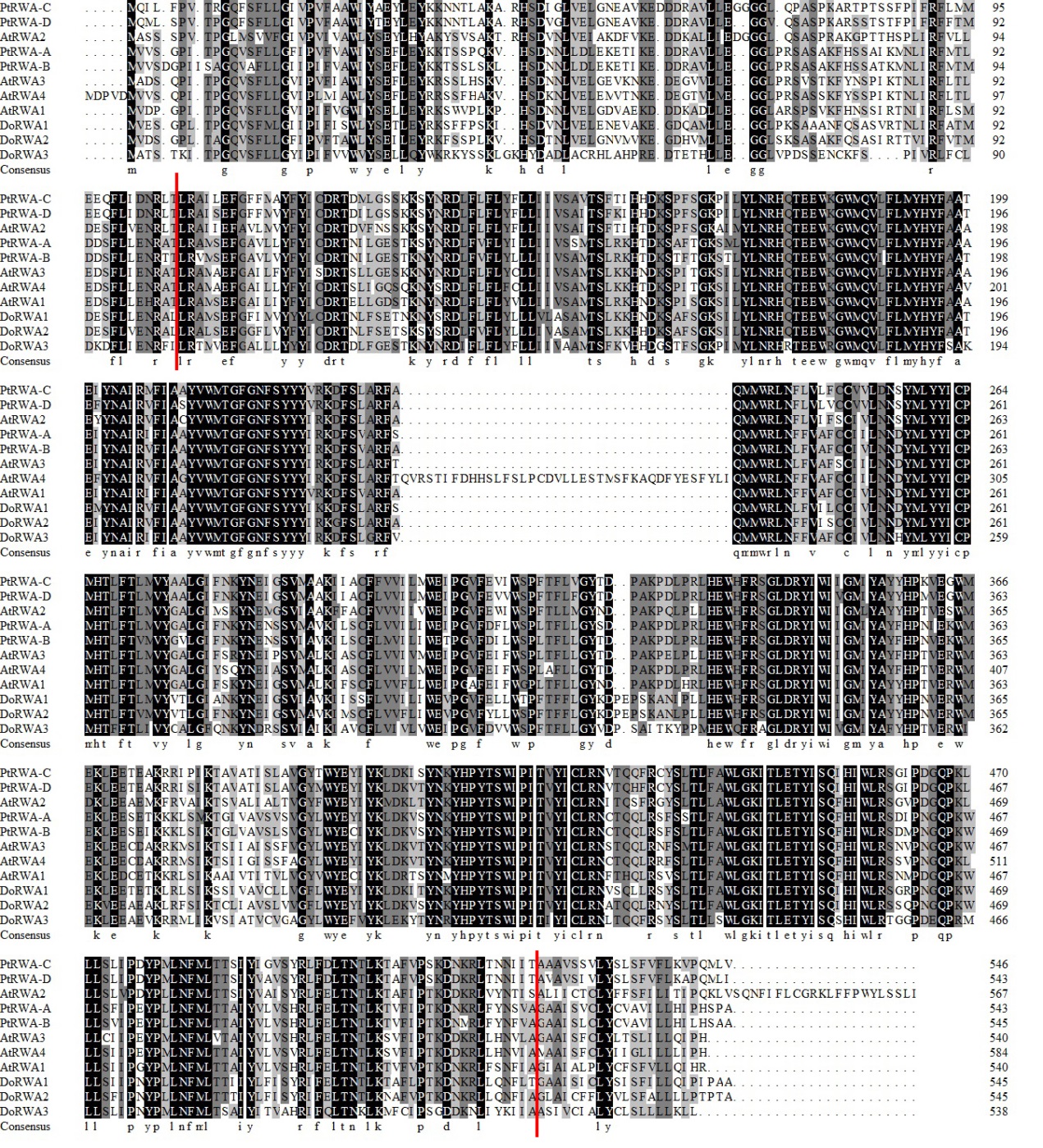


**Figure S1**


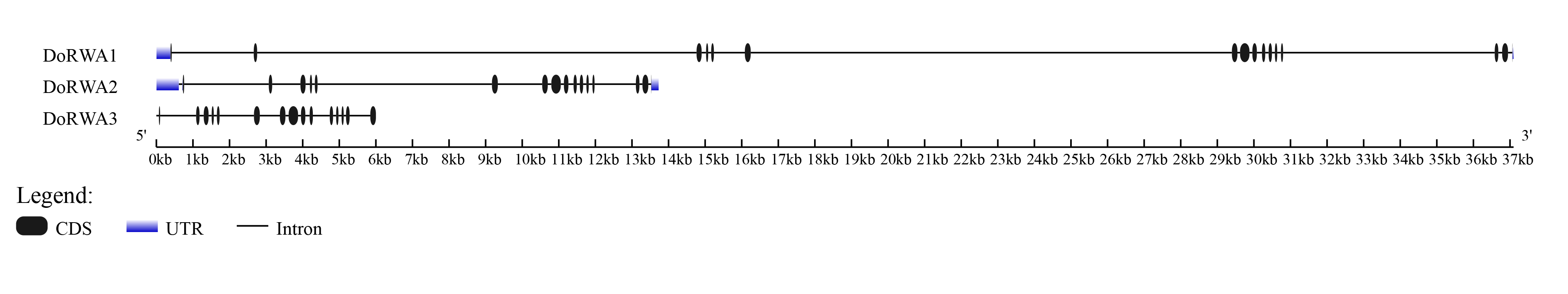


**Figure S2**


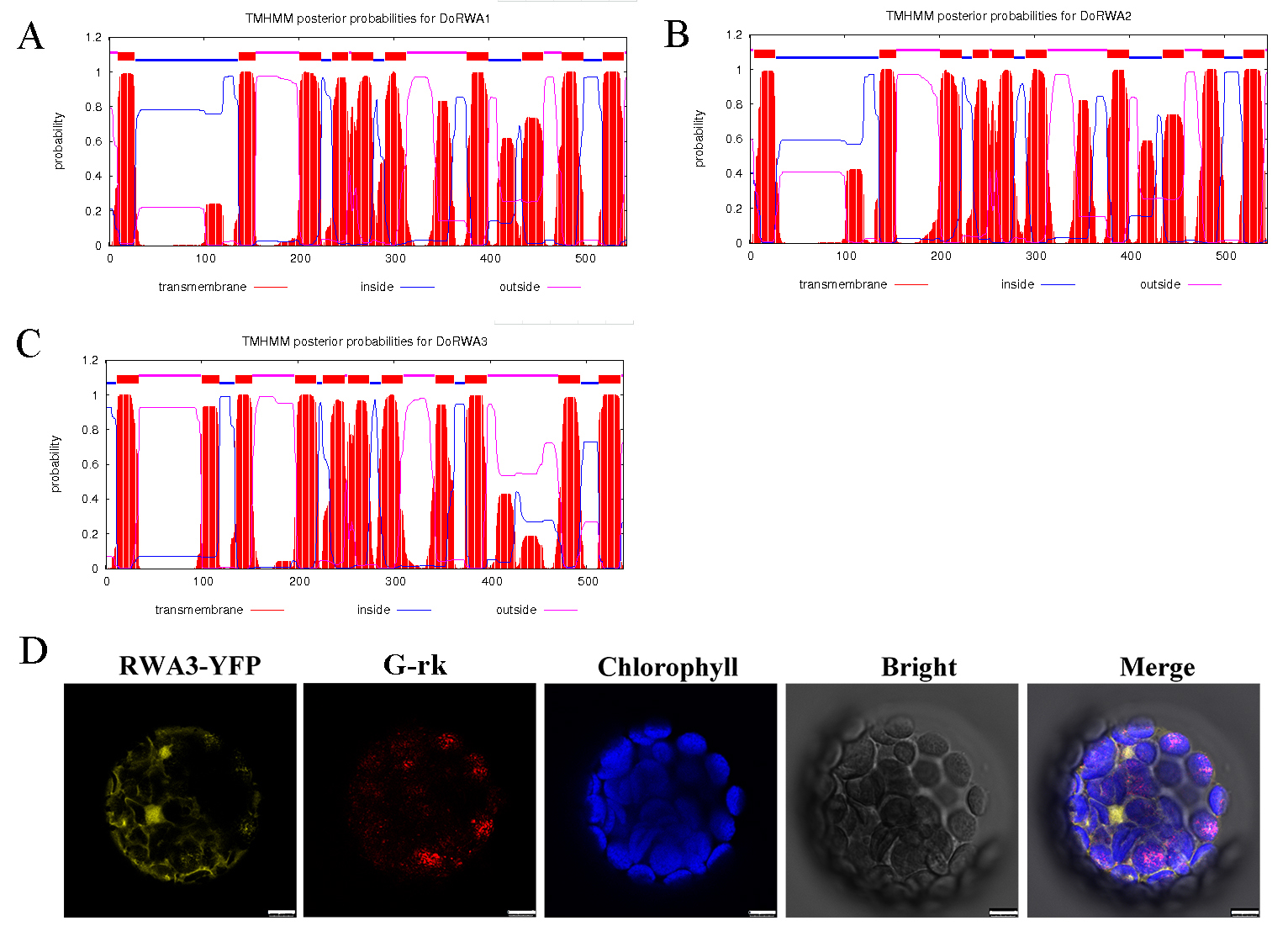


**Figure S3**


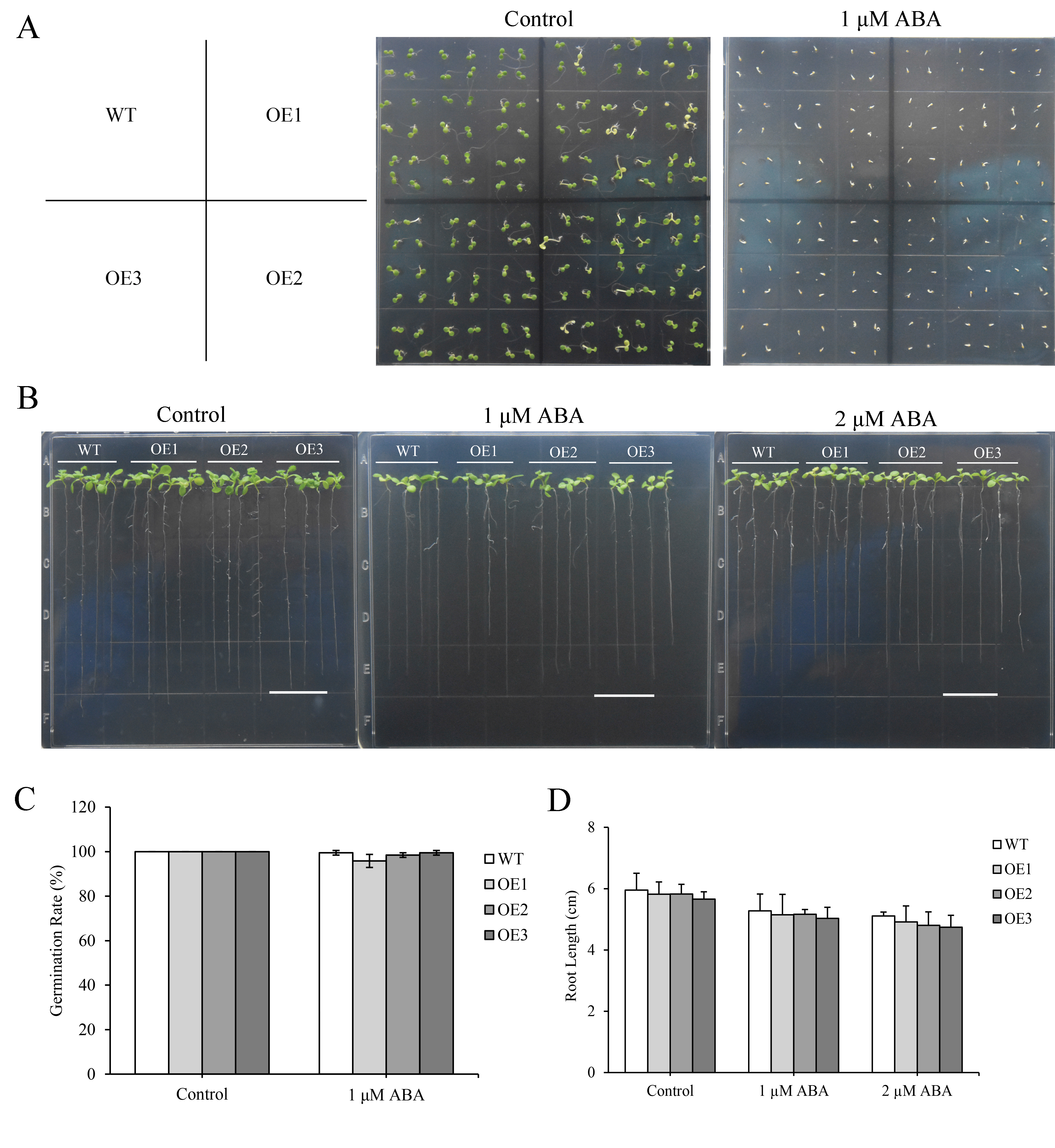


**Figure S4**
